# Organoids in Haematologic Research: Advances and Future Directions

**DOI:** 10.1111/cpr.13806

**Published:** 2025-01-26

**Authors:** Liangzheng Chang, Lu Li, Yuling Han, Hui Cheng, Liuliu Yang

**Affiliations:** ^1^ State Key Laboratory of Experimental Hematology, National Clinical Research Center for Blood Disease, Haihe Laboratory of Cell Ecosystem Institute of Hematology & Blood Diseases Hospital, Chinese Academy of Medical Sciences & Peking Union Medical College Tianjin China; ^2^ Tianjin Institute of Health Science Tianjin China; ^3^ Key Laboratory of Organ Regeneration and Reconstruction, State Key Laboratory of Stem Cell and Reproductive Biology Institute of Zoology, Chinese Academy of Sciences Beijing China; ^4^ Chinese Academy of Sciences Institute for Stem Cell and Regeneration Beijing China; ^5^ Beijing Institute for Stem Cell and Regenerative Medicine Beijing China

## Abstract

Organoid technology, as a revolutionary biomedical tool, has shown immense potential in haematological research in recent years. By using three‐dimensional (3D) cell culture systems constructed from pluripotent stem cells (PSCs) or adult stem cells (ASCs), organoids can highly mimic the characteristics of in vivo organs, thereby offering significant potential for investigating human organ development, disease processes and treatment strategies. This review introduces the development of organoids and focuses on their progress in haematological research, including haematopoietic‐related organoids, immune‐related organoids and organoids used for studying blood system diseases. It discusses the prospects, challenges and future outlook of organoids in the field of haematology. This review aims to provide the latest advancements and future directions of organoid technology in haematological research, offering references and insights into further exploration in this field.

## Graphical abstract

This review focuses on the advancements in organoid technology and its transformative role in haematological research, particularly in the modelling of haematopoietic tissues, blood diseases and drug development. It also offers an outlook on the future prospects of organoids in personalised medicine and the study of disease mechanisms.

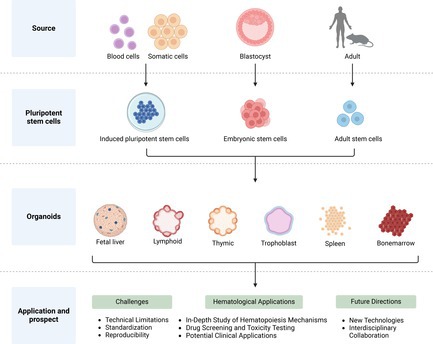

## 1 Introduction

In recent years, organoids have become a revolutionary tool in biomedical research, providing unprecedented opportunities for studying human development, diseases and treatments [1]. Using three‐dimensional (3D) cell culture systems constructed from pluripotent stem cells (PSCs) or adult stem cells (ASCs), organoids can self‐organise into miniaturised and simplified versions of organs, closely mimicking the characteristics of in vivo organs [2, 3, 4]. This ability to replicate the key structures and functions of real organs makes organoids extremely valuable in many fields, including haematology [5].

The blood system is a crucial part of the human body, playing a vital role in maintaining homeostasis, fighting diseases and facilitating tissue repair. Abnormalities in the blood system can lead to various diseases such as anaemia, leukaemia, lymphoma and haemorrhagic diseases. These conditions not only severely affect patients' quality of life but also be life‐threatening. Therefore, a deep understanding of the physiological and pathological processes of the blood system is crucial for the prevention, diagnosis and treatment of blood‐related diseases.

Traditional in vitro research methods and animal models often have limitations when studying the complexity of the blood system and related diseases [6]. Two‐dimensional (2D) cell culture systems lack tissue structure and microenvironment, failing to fully mimic the complex cell interactions in vivo [7]. Animal models, although providing an in vivo environment, often produce results that do not apply to humans due to species differences [8]. Therefore, developing research tools that can better simulate the human blood system has become imperative.

The development of organoid technology has brought new hope to haematological research [9]. These 3D culture systems can not only display more realistic cell arrangements and tissue structures but also simulate the microenvironment of the blood system, providing more accurate research models [10]. Currently, organoids have shown significant application prospects in the research of haematopoiesis, immune regulation, leukaemia and other blood system diseases.

In this review, we briefly introduced the development of organoids and focused on their progress in haematological research, including haematopoietic‐related organoids, immune‐related organoids and organoids used for studying blood system diseases. Finally, we explored the future directions of organoids in haematological research, examining their potential in personalised medicine, drug screening and disease mechanism studies.

## 2 Development of Organoids

Organoid growth is a process involving initial cell aggregation, proliferation and differentiation [10, 11]. 3D organoid cultures can originate from various sources such as embryonic stem cells (ESCs), ASCs, induced pluripotent stem cells (iPSCs) and tissue fragments [12, 13]. They represent the tissue structure and cell specificity found in the original tissues or tumours [14].

**FIGURE 1:**
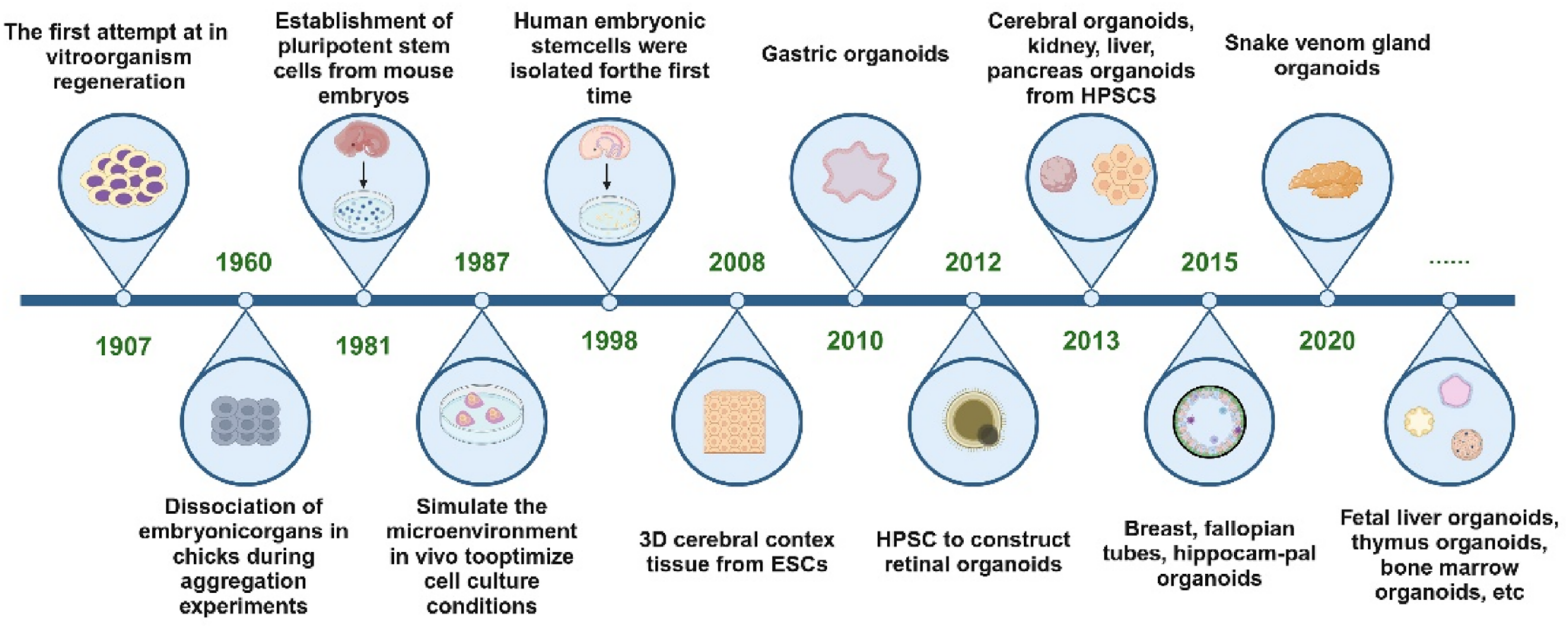
The chronological history of the development of organoids.

Since the early 20th century, the continuous development of cell culture technology has drawn widespread attention to 3D cell culture [15]. The origin of organoids can be traced back to 1907 when H.V. Wilson conducted in vitro regeneration studies on sponges. They discovered that dissociated sponge tissues could self‐organise and regenerate into a fully functional organism [16]. In 1960, researchers dissociated various organs from chick embryos and found that these could self‐organise into corresponding organs after in vivo transplantation [17]. This discovery laid the foundation for the development of organoid technology and the theory of cell self‐organisation. Stem cell research began to flourish in 1981 when PSCs were first isolated and established from mouse embryos [18]. In 1987, Dr. Li and colleagues optimised cell culture conditions by simulating the in vivo microenvironment. They found that mammary carcinoma epithelial cells could form 3D ducts and lumens from extracellular matrix extracts [19]. Additionally, alveolar type II epithelial cells maintained their differentiation in the presence of an ECM matrix [20]. However, it was not until 1998 that James Thomson first successfully isolated human ESCs [21]. In 2008, Eiraku and colleagues generated cortical tissues from ESCs using a 3D aggregation culture method [22]. This sparked a surge in stem cell research and clinical applications, transitioning organoid research from 2D to 3D. In 2009, Dr. Hans Clevers and colleagues discovered that G protein–coupled receptor 5 (Lgr5) is exclusively expressed in cycling columnar cells at the base of crypts. They successfully constructed 3D intestinal organoids from single stem cells in Matrigel [23, 24]. This provided a foundation for organoid research, sparking a surge of interest in the field (Figure 1).

The major achievements in organoid development have occurred mainly in the past decade. Currently, 3D organoid culture technology has successfully produced numerous tissue‐like organs with key physiological structures and functions, such as kidneys [25], liver [26], lungs [27], intestines [28], brain [29], prostate [30], pancreas [31] and retina [32, 33, 34]. In 2012, Nakano and colleagues demonstrated that human pluripotent stem cells (hPSCs) could self‐organise into optic cup organoids in a 3D construct [33, 35]. In 2013, Lee and colleagues found that endothelial cells and adult bronchioalveolar stem cells could generate lung organoids in a 3D co‐culture system [36]. In 2014, it was discovered that isolated and recombined mouse embryonic kidney stem cells could form kidney organoids [25]. Intestinal organoids can be generated from iPSCs in vitro [28]. In 2015, organoids of the mammary gland, fallopian tube and hippocampus were successively generated [37, 38, 39]. In 2020, venom gland organoids were produced [40]. From self‐organisation to stem cells, and then to organoid technology, organoids have opened a new chapter in regenerative medicine.

**FIGURE 2:**
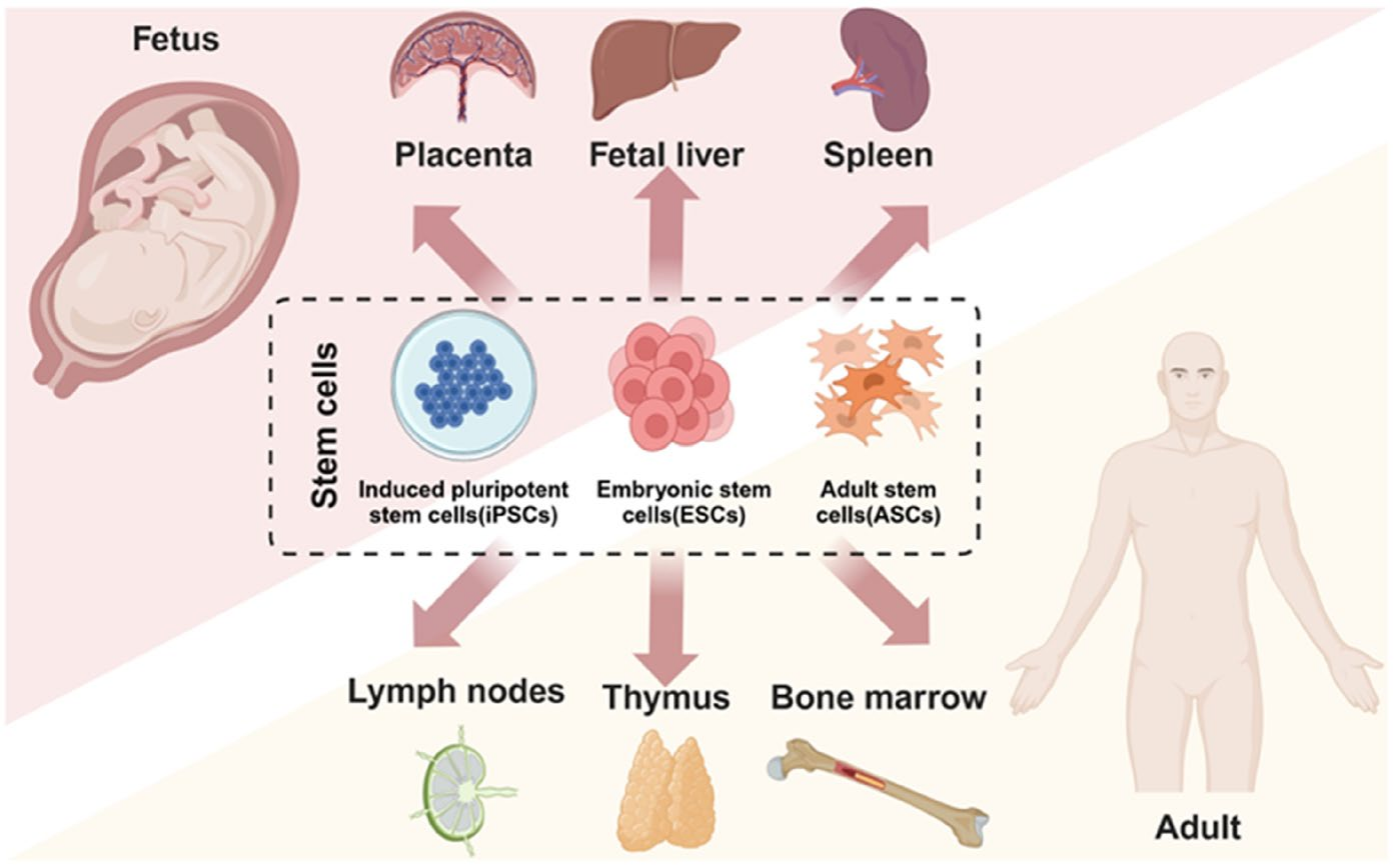
Organoids in haematological research.

**FIGURE 3:**
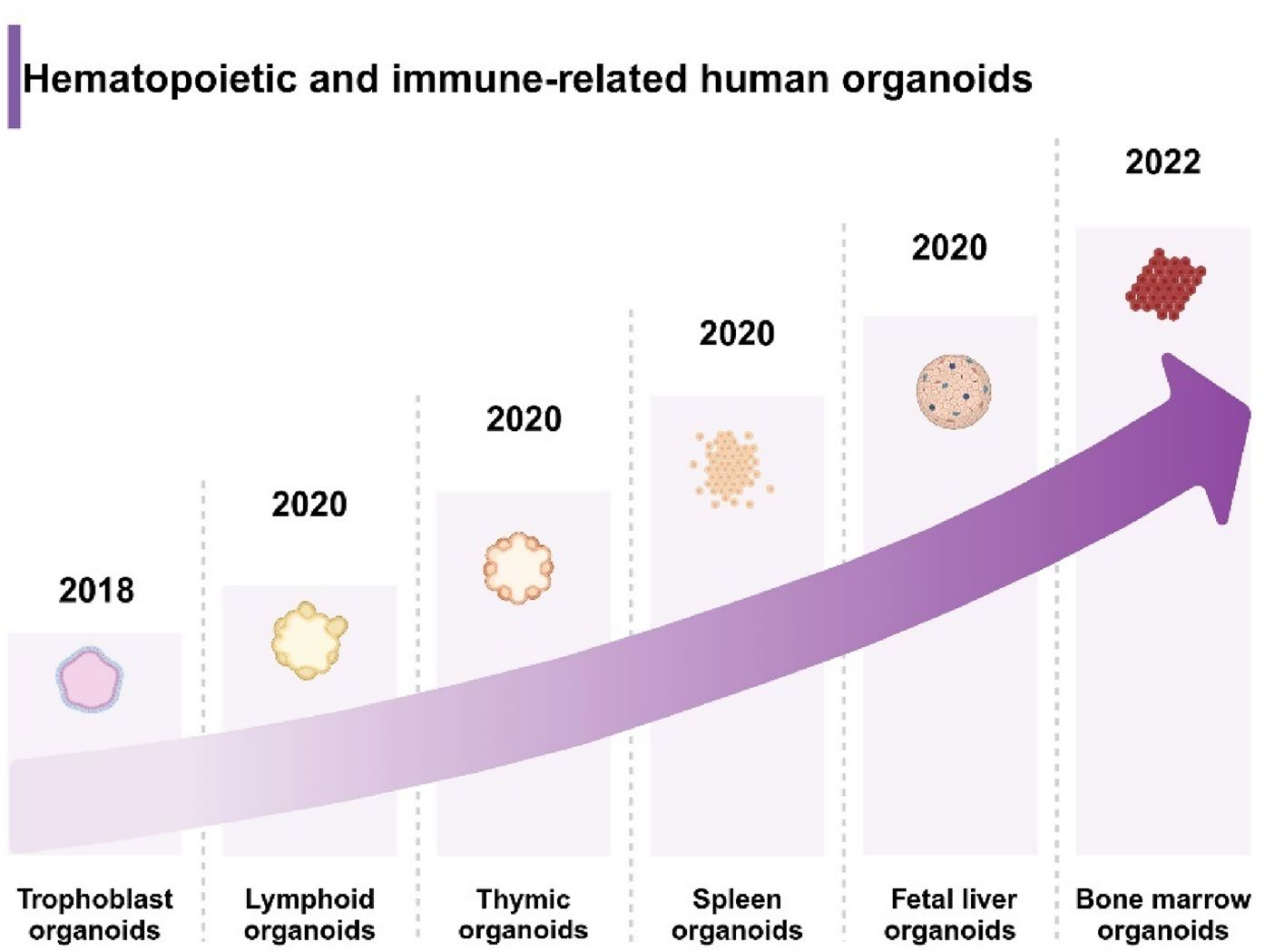
Timeline of organoid development in haematological research.

## 3 Organoids in Haematological Research

### 3.1 Bone Marrow Organoids

Increasing experimental evidence suggests that bone marrow, particularly the haematopoietic niche within it, plays a crucial role in the function of haematopoietic stem cells (HSCs). The haematopoietic niche is a complex microenvironment composed of various cell types, extracellular matrix and secreted signalling molecules that support and regulate the self‐renewal and differentiation of HSCs. Additionally, disturbances in the bone marrow haematopoietic niche promote the development and spread of haematologic malignancies. Therefore, research on bone marrow is essential for understanding the human haematopoietic system and its related diseases.

The development of bone marrow organoids began in the early 21st century, with scientists attempting to mimic the 3D structure and function of bone marrow in vitro. Initial studies focused on using PSCs and ASCs to construct functional bone marrow microenvironment models in vitro [41, 42]. In 2011, Tormin and colleagues successfully constructed a 3D bone marrow microenvironment model in vitro using mesenchymal stem cells that could support HSC growth, marking a significant breakthrough in bone marrow organoid research [43]. Sayo and colleagues used 3% methylcellulose to culture mouse bone marrow tissue in vitro, successfully forming bone marrow aggregates. Although the cell density was slightly lower, organoid sections resembled intact bone marrow tissue. Additionally, staining for CD68, PDGFRa and CXCL12 in macrophages and mesenchymal cells confirmed the reconstruction of bone marrow tissue [44]. Currently, human iPSCs have been successfully used to construct bone marrow organoids, and single‐cell RNA sequencing has verified the heterogeneity of cells within the organoids [45]. Additionally, xenotransplantation in mice has revealed the lymphoid potential of iPSC‐derived bone marrow organoids (iPSC‐BMOs) [46]. iPSC‐BMOs also support the implantation and survival of malignant blood tumour cells from patients, aiding the exploration of personalised treatment plans for patients with haematologic malignancies. Furthermore, after TGF‐β stimulation and bone marrow fibrosis implantation, organoids underwent fibrosis, whereas cells from healthy donors did not, validating organoids as a powerful tool for studying malignant cells and their interactions in a human bone marrow–like environment [41].

### 3.2 Foetal Liver Organoids

During embryonic development, the liver plays a crucial role in haematopoiesis. The foetal liver is the primary site for the development and expansion of HSCs. The haematopoietic microenvironment in the liver provides the necessary signals and support, allowing HSCs to proliferate and differentiate into mature blood cells. This process begins around the third week of foetal development and rapidly increases from the fifth to the tenth week [47, 48]. Additionally, the liver participates in extramedullary haematopoiesis during both foetal and adult periods, generating blood cells outside the bone marrow. This is particularly important under certain pathological conditions, such as bone marrow dysfunction or specific blood diseases [49].

Constructing liver organoids, particularly foetal liver organoids, aids in the study of early human haematopoiesis and extramedullary haematopoiesis under pathological conditions. Hendriks et al. successfully established culture conditions that favour the long‐term expansion of human foetal liver cells as organoids. They used CRISPR‐Cas9 technology to perform (multi) gene knockouts and gene knock‐ins in the human foetal liver organoid system, applicable for observing and intervening in foetal liver development and haematopoietic functions [50]. However, human foetal liver cells are often difficult to obtain due to ethical constraints and other factors. Mun's team developed a method to efficiently and reproducibly generate functionally mature human liver organoids from PSCs, aiding in the understanding of liver development and regeneration and providing insights into haematopoietic development in the liver [51]. Unlike Matrigel‐based liver organoids, new liver organoids generated through micropatterning technology can form bioengineered foetal liver organoids with uniform morphology, defined size and position in a reproducible and high‐throughput manner in multi‐well plates. The organoids, crafted through this method, meticulously mirror pivotal developmental aspects of the foetal liver, encompassing the expression of genes and proteins unique to the foetal liver, the accumulation of glycogen, the buildup of lipids and the secretion of proteins [52]. Another study reported that self‐assembled, matrix‐free iPSC‐derived organoids developed in a rotating wall vessel (RWV) exhibit stronger hepatocyte‐specific functions than those formed on Matrigel. RWV liver organoids maintain a sustained function in long‐term culture and express a range of mature functional genes at levels comparable to adult liver while retaining some foetal characteristics, making them more suitable for studies on hepatic haematopoiesis [53].

### 3.3 Spleen Organoids

The spleen plays multiple roles in haematopoiesis, particularly in regulating and supporting the production and maintenance of blood cells. Firstly, the spleen is an important blood storage organ, capable of storing red blood cells, platelets and white blood cells and quickly releasing them when the body needs to maintain blood balance [42, 54]. Additionally, the spleen has haematopoietic functions during foetal development, producing red blood cells, white blood cells and megakaryocytes. In adults, this function may partially resume under certain conditions, such as bone marrow dysfunction [55]. Most importantly, the spleen plays a crucial role in removing aged or damaged red blood cells and filtering pathogens and immune complexes from the blood, which is essential for maintaining blood health and the normal function of the immune system [54, 55]. The integration of these functions makes the spleen an indispensable organ in the haematopoietic system, crucial for understanding and treating various blood diseases [42, 54].

Currently, research on spleen organoids is gradually developing, demonstrating their potential in regenerative medicine and disease modelling. For example, a study generated tissue‐engineered spleens by implanting spleen organoid units into immunodeficient mice, successfully recreating the red and white pulp structures and immune functions of the spleen. These tissue‐engineered spleens were able to reduce red cell inclusions following splenectomy, indicating their potential to restore immune function [56]. This study successfully applied organoid technology to restore spleen structure, providing possibilities for further research on innate and pathological haematopoiesis in the spleen.

### 3.4 Placenta Organoids

In terms of haematopoiesis, the placenta is considered one of the important haematopoietic organs during the foetal period [57]. Studies have shown that the placenta not only contains a large number of HSCs but also provides a unique microenvironment that supports the proliferation and differentiation of these cells [58]. The haematopoietic function of the placenta varies at different stages of embryonic development. In the early embryonic period, the placenta is one of the main sites of haematopoiesis, supporting the generation and differentiation of primitive HSCs [59]. As the embryo develops, the haematopoietic function of the placenta is gradually replaced by the liver and bone marrow, but it remains an important reservoir of HSCs [60]. HSCs in the placenta have high self‐renewal capacity and multipotency, making them crucial for the development of the foetal and neonatal blood systems [61]. Moreover, the placenta provides an immune‐privileged environment during haematopoiesis, protecting the foetus from attacks by the maternal immune system [62].

Placental organoids have made significant progress in haematopoietic research, providing new platforms and perspectives for understanding the role of the placenta in haematopoiesis. Through engineered placental organoids, researchers can generate placenta‐like structures with endogenous vascular cells from human iPSCs, successfully mimicking the microenvironment of the placenta [63]. Additionally, functional multicellular organoids generated from human placental villi exhibit structures and functions similar to the natural placenta, providing a reliable model for studying the role of the placenta [64]. The establishment and differentiation of long‐term trophoblast organoids further provides an experimental model to investigate human placental development and function [65]. Using single‐cell assessment techniques, researchers analysed primary and stem cell–derived human trophoblast organoids, finding them to be effective platforms for simulating the placenta, aiding in the in‐depth study of the placenta [66]. Self‐renewing trophoblast organoids recreate the developmental programmes of the early human placenta, providing valuable models for studying early placental development and haematopoiesis [67]. Trophoblast organoids derived from stem cells are capable of replicating the developmental processes of the human placenta and are prone to infection by newly emerging pathogens. This capability opens up novel avenues for investigating the placental contributions to blood formation and immune system protection [68]. Additionally, trophoblast organoids are used as platforms to study maternal–foetal interactions, revealing how the placenta regulates haematopoiesis and immune functions during pregnancy [69]. Trophoblast organoids with physiological polarity successfully simulate the structure and function of the placenta, providing new tools for studying the specific mechanisms of placental haematopoiesis [70]. Organoid models based on trophoblast stem cells help study the role of the human placental barrier in protecting the foetus [71]. Mouse trophoblast organoid models, using CRISPR‐Cas9 screening technology, help study the function of trophoblast cells in placental development [72].

Researchers have begun exploring the use of placenta‐derived HSCs for bone marrow transplantation and gene therapy to treat various blood disorders. Due to the low immunogenicity of placenta‐derived HSCs, the risk of rejection during transplantation is lower, offering new hope for clinical treatments [73].

### 3.5 Lymph Node–Like Organoids

Lymph nodes play a crucial role not only in immune responses but also in the study of haematologic malignancies. Recent studies have found that the microenvironment of lymph nodes significantly impacts the development and progression of haematologic malignancies such as lymphoma and leukaemia. For example, studies have shown that stromal cells and immune cells in lymph nodes can promote the survival and proliferation of tumour cells by secreting various cytokines and chemokines [74]. Additionally, lymph nodes play an important role in the homing and differentiation of HSCs. Research indicates that high endothelial venules (HEVs) in lymph nodes can attract circulating HSCs to migrate to the lymph nodes and differentiate, thereby secreting chemokines CCL19 and CCL21 [75, 76]. This mechanism is crucial for understanding the behaviour of HSCs and developing new stem cell therapy strategies.

In recent years, lymph node organoids have made significant progress in haematological research, providing new platforms for understanding immune functions, immune diseases and immunotherapies. By generating synthetic lymphoid tissue–like organoids in mice, researchers can simulate the microenvironment of lymph nodes, allowing the study of the role of lymphoid tissues in immune responses [77]. These organoids exhibit structures and function similar to natural lymphoid tissues, providing important tools for studying in vivo immune responses. Immunoassays conducted on human lymphoid micro‐organoids in vitro demonstrate that these organoids can be used to assess immune responses and test the efficacy of new immunotherapeutic drugs [78]. Significant progress has been made in exploring immune functions, immune diseases and immunotherapies through organoid research. Organoid technology enables researchers to study complex immune processes in a controlled environment, leading to a deeper understanding of the immune system's mechanisms and pathological processes [79]. This provides a theoretical basis and experimental models for developing new immunotherapies. Rapidly generated HEV organoids derived from hPSCs demonstrate the ability to form ectopic lymphoid tissues in vivo [80]. These organoids can mimic the functions of HEVs, providing new models for studying the homing and differentiation of HSCs in lymph nodes. This advancement offers new perspectives for understanding the behaviour and function of HSCs in the lymph node microenvironment. Studying the dynamics of stimulation and inhibition on neural and lymphoid organoids reveals the responses and adaptation mechanisms of these tissues under different conditions [81]. This research helps in understanding the functional changes in lymphoid tissues in health and disease, providing important information for developing targeted treatment strategies. Therapeutic regeneration studies on lymphoid organoid transplantation show that these organoids can restore the function of lymphoid and immune cells [82]. By transplanting organoids, researchers observed significant improvements in the immune function of recipients, demonstrating the potential of organoids in treating immunodeficiency and other immune‐related diseases.

### 3.6 Thymus Organoids

The function of the thymus is crucial in the haematopoietic system, especially in the generation of T cells and the establishment of immune tolerance [83]. Studies have shown that various cells and molecular signals in the thymic microenvironment play key roles in the selection and maturation of T cells. For example, cytokines and chemokines secreted by thymic epithelial cells, such as IL‐7 and CCL25, are critical for the survival and migration of T‐cell precursors [84, 85]. Thymus transplantation can effectively rebuild T‐cell immune function, particularly in patients undergoing bone marrow or stem cell transplantation [86]. In studies on thymic degeneration and immunosenescence, scientists have found that thymic function significantly declines with age, leading to reduced T‐cell production and weakened immune function [87]. This process is believed to be related to functional changes and structural disruption of thymic stromal cells. Therefore, researchers are developing new intervention strategies, such as using growth factors, cell therapy and gene editing technologies, aiming to delay or reverse thymic degeneration and improve immune function in the elderly [88].

In recent years, artificial thymus organoids have shown great potential in T‐cell differentiation and the treatment of patients with T‐cell lymphopaenia. These organoids simulate the microenvironment of the natural thymus, providing a reliable platform for the generation and maturation of T cells. For example, artificial thymus organoids are used to study the T‐cell differentiation process, providing valuable research tools for patients with severe T‐cell lymphopaenia [89]. By transplanting iPSC‐derived thymus organoids into humanised mice, researchers successfully constructed new T‐cell compartments, providing a new model for studying human T‐cell development [90]. Functional thymic epithelial organoids derived from adult mouse thymus have shown the ability to maintain T‐cell development in vitro, providing a stable experimental system for studying thymic epithelial cell functions [91]. Using tissue‐engineered thymus organoids, scientists can efficiently generate human T cells, offering a new hope for the treatment of T‐cell‐related diseases [92]. Additionally, using gene editing technology, researchers successfully rescued T‐cell development in RAG2‐deficient iPSCs within artificial thymus organoid systems, demonstrating the great potential of gene editing in correcting genetic defects and restoring immune function [93]. Generating functional thymus organoids from hPSCs provides a powerful model for studying thymus development and T‐cell differentiation [94]. These organoids can generate mature T cells and exhibit structures and functions similar to those of the natural thymus [95]. By generating mature T cells in artificial thymus organoids, researchers further validated the potential of this model in studying the development of human HSCs and precursor cells [96]. Applying natural thymic extracellular matrix to in vivo thymus organoids significantly increased T‐cell production and promoted thymic epithelial cell differentiation in vitro [97]. This research demonstrates the potential of natural matrices in optimising organoid functions. Additionally, by culturing and regenerating thymic tissue in vitro, scientists are exploring ways to improve immunodeficiency and promote immune reconstitution. For instance, bioengineered thymus organoids have successfully restored thymic function, offering new therapeutic strategies for patients with impaired immune function [98, 99] (Figures 2 and 3).

### 3.7 Organoids in Haematological Diseases

The complexity and heterogeneity of haematologic malignancies present significant challenges for their diagnosis and treatment. Tumour organoid technology faithfully replicates the genetic and phenotypic characteristics of tumours in vitro. This advancement facilitates the study of tumour mechanisms and supports personalised therapies and drug screening. In the field of haematologic malignancies, tumour organoids have proven invaluable for elucidating signalling pathways within tumours and their microenvironments, significantly advancing research on drug resistance and the development of new therapies.

#### 3.7.1 Lymphoma

Currently, there is a lack of sufficient ex vivo lymphoma models to study the factors influencing its development and the microenvironment in which it resides [100]. Research using tumour organoids has revealed that the B‐cell receptor (BCR) signalling pathway plays a pivotal role in the progression of diffuse large B‐cell lymphoma (DLBCL). Furthermore, T cells in the tumour microenvironment modulate histone modifications, such as H3K9me3, which alter the efficiency of BCR signalling and contribute to therapeutic resistance [101]. This phenomenon, where the microenvironment regulates gene expression and signalling, has been further validated in other lymphoma organoid studies. Sha et al. demonstrated that in DLBCL, microenvironmental signals enhance BCR and TLR pathway activities, weakening the efficacy of BCR‐targeted therapies [102]. Additionally, Vidal‐Crespo et al. used organoid models to demonstrate the significant antitumor activity of the anti‐CD38 antibody Daratumumab in non‐Hodgkin lymphoma, further validating the utility of organoids in drug screening [103]. Tumour organoids also provide robust support for modelling the tumour microenvironment of haematologic malignancies in vitro. Kastenschmidt et al. demonstrated that follicular lymphoma (FL) organoids can maintain a stable microenvironment in long‐term cultures and accurately mimic T‐cell‐mediated immune responses, providing an experimental platform for the development of novel immunotherapies [104]. Meanwhile, the Faria team developed patient‐derived lymphoma spheroid models, which successfully captured the immune characteristics of FL and demonstrated potential for testing immunotherapies [105]. In addition to modelling the cellular biology of haematologic malignancies, organoids can also aid in understanding the dependency of tumour cells on their microenvironment. Tian et al. constructed an integrin‐specific hydrogel organoid system that revealed the critical role of integrin signalling in tumour cell proliferation and drug resistance, showcasing how organoid systems can be used to study the regulation of the extracellular matrix in the microenvironment [100].

#### 3.7.2 Multiple Myeloma

Multiple myeloma is a haematologic malignancy derived from differentiated B cells, primarily located in the bone marrow niche. The challenge in treatment lies in the uniqueness of its tumour microenvironment, highlighting the advantages of organoid models [106]. The challenge in treating multiple myeloma lies in the uniqueness of its tumour microenvironment. Conventional 2D cultures and commonly used established multiple myeloma cell lines cannot replicate the in vivo microenvironment, which highlights the advantages of 3D organoid models [107]. Bone marrow organoids can simulate the BM microenvironment, making tumour cell behaviour more physiological and providing insights into the pathophysiology of multiple myeloma. This helps to better understand tumour proliferation, resistance mechanisms and the application of novel immunotherapies, such as antibody therapies, engineered cells and immune modulators, in preclinical research. In multiple myeloma studies, Wei et al. used organoid models derived from bone marrow biopsy cells to further elucidate the key role of histone demethylase LSD1 in the pathogenesis of multiple myeloma [108]. However, research on multiple myeloma organoids is still limited, with significant potential for future development.

#### 3.7.3 Leukaemia

In the field of leukaemia, it is well known that culturing non‐solid leukaemia cells from patients in vitro is extremely challenging. However, the advent of organoid technology has greatly improved this situation. The successful establishment of bone marrow organoids has made it possible to culture and maintain malignant cells from patients with acute lymphoblastic leukaemia and myeloid leukaemia (ALL) in vitro [41]. Additionally, the application of organoid technology has expanded into other areas, such as leukaemia research related to the central nervous system (CNS). Gebing et al. developed a co‐culture model of brain organoids and ALL cells, revealing the critical role of the AP‐1 signalling pathway in CNS leukaemia progression, demonstrating that organoids can effectively model the complexity and multisystem involvement of haematologic malignancies [109].

In addition to haematologic malignancies, organoid technology has also been applied to the study of other blood disorders, such as anaemia. By constructing anaemia models, researchers can simulate red blood cell production disorders and study gene mutations and pathological mechanisms associated with anaemia [110].

## 4 Applications of Organoids in Haematology

### 4.1 In‐Depth Study of Haematopoiesis Mechanisms

Organoid technology aids in deepening the understanding of blood generation mechanisms. By constructing 3D human bone marrow organoids, researchers can replicate normal and abnormal haematopoiesis processes, providing new insights into understanding haematopoiesis mechanisms [110]. These organoids can simulate the complex cell–cell interactions in the bone marrow, including interactions between HSCs, stromal cells and immune cells [41]. Using 3D‐printed bone marrow organoids with specific attributes can ensure the recreation of elements of the microenvironment, including lymphocyte populations, allowing the study of immune cell dynamics, stress responses and cell–cytokine interactions [110]. Additionally, using static organoids and porous scaffolds, researchers have successfully expanded HSCs and validated their function in mouse models, further demonstrating the application prospects of organoid technology in haematopoiesis research [111].

While bone marrow organoids simulate and study intramedullary haematopoiesis, research into extramedullary haematopoiesis is equally crucial for understanding blood generation. The liver and spleen play roles in extramedullary haematopoiesis during foetal development and in certain postnatal conditions. By constructing 3D organoids containing liver sinusoidal endothelial cells, the haematopoietic environment of the liver can be modelled. These organoids support blood cell formation and offer a novel model for studying hepatic haematopoiesis, particularly under pathological conditions such as bone marrow failure [112]. Furthermore, foetal liver organoids, built using human foetal liver cells, enhance the understanding of embryonic liver haematopoiesis [50]. Spleen organoids effectively recreate the splenic microenvironment, revealing how HSCs are maintained and differentiated within this niche [113]. Organoid technology thus allows for a comprehensive study of blood generation mechanisms, including both intramedullary and extramedullary processes, deepening our understanding of haematopoiesis and providing valuable insights for developing new therapies.

### 4.2 Drug Screening and Toxicity Testing

Organoid technology shows immense potential in drug screening and toxicity testing. By constructing 3D human bone marrow organoids, researchers can replicate the complex bone marrow microenvironment in vitro, enhancing the precision and efficacy of drug screening [41]. These organoid models not only recreate normal and abnormal haematopoiesis but also assess the specific effects of drugs on different cell types, enabling the selection of candidates with high efficacy and low toxicity [110]. Additionally, using organoid models for toxicity testing allows for better prediction of potential adverse effects in humans, thereby improving the safety and success rate of drug development [111]. For instance, researchers have successfully evaluated the efficacy and toxicity of a series of anti‐leukaemia drugs using bone marrow organoid models, providing reliable data support for preclinical drug development [114]. These advancements indicate that organoid technology has broad application prospects in drug screening and toxicity testing, significantly accelerating drug development and improving clinical translation rates [115].

### 4.3 Potential Clinical Applications

**FIGURE 4:**
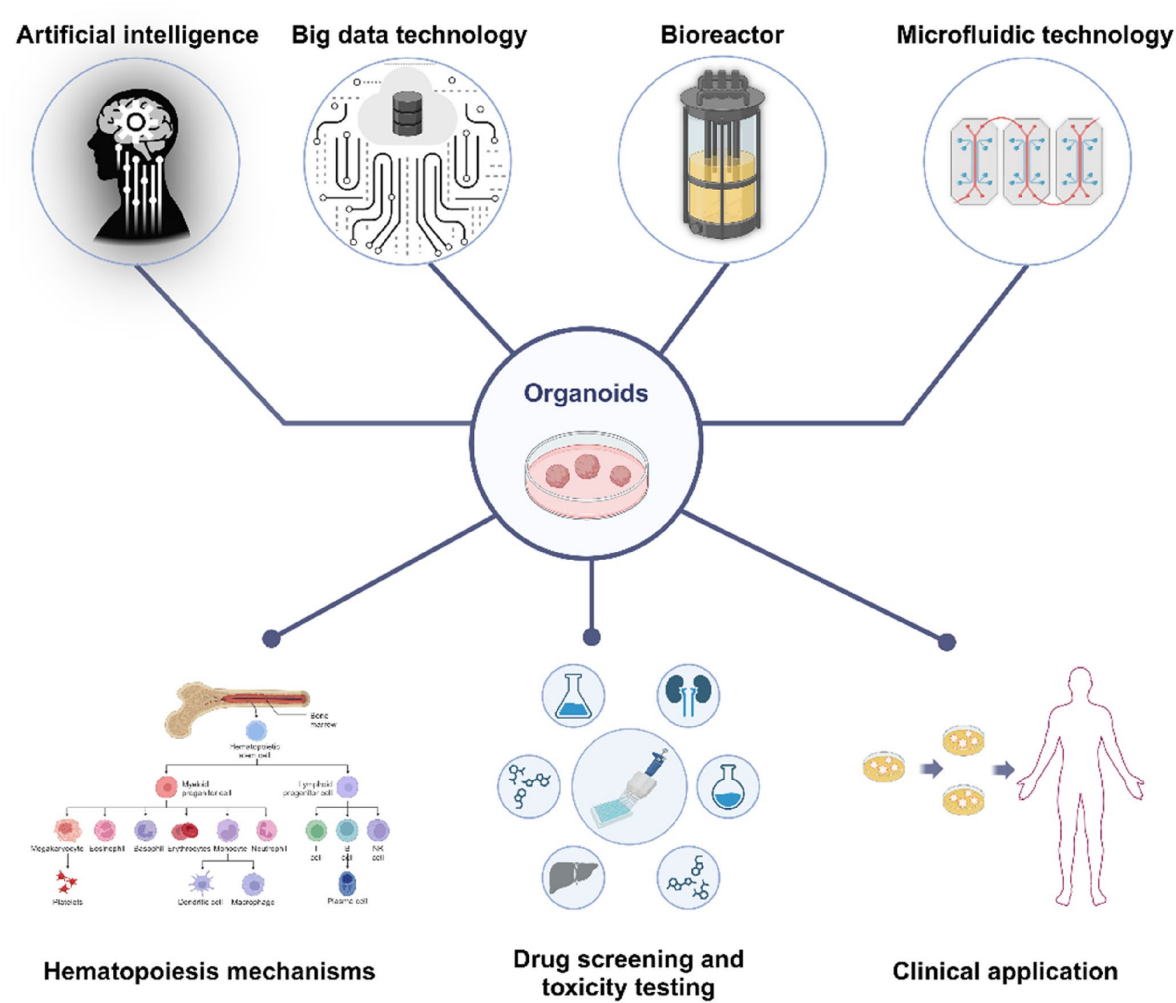
The future development of organoids and their application in haematology.

HSC transplantation is used to treat various haematologic disorders, including leukaemia and multiple myeloma [116]. Studies showed that HSCs can restore haematopoiesis and, under certain conditions, promote the repair and regeneration of non‐haematopoietic tissues such as the liver, heart and brain [117]. HSCs can replace the entire blood system and induce immune tolerance during transplantation, reducing immune rejection [118]. However, obtaining suitable HSCs for treatment is often challenging due to human leukocyte antigen matching requirements [119]. Organoid technology offers a new hope for the large‐scale production of HSCs. By creating an in vitro environment conducive to HSC proliferation, organoids facilitate their maintenance and expansion. Studies have shown that multipotent RAG1^+^ progenitor cells can be derived directly from haematopoietic endothelial cells within organoids generated from hPSCs [120]. Serum‐free and feeder‐free organoid induction methods have successfully derived definitive HSCs from human ESCs [121]. Notably, self‐organising yolk sac organoids derived from human iPSCs have expanded multipotent haematopoietic progenitor cells, enhancing the feasibility of bulk production for clinical applications [122]. Furthermore, the implantation of haematopoietic organoids is also a promising area of investigation. Researchers have demonstrated that implanting bone organoids into mice increases the levels of HSCs and that cells derived from these organoids can quickly and effectively reconstruct damaged peripheral and solid immune organs in irradiated mice, providing strong evidence for therapeutic organoid transplantation [123]. Moreover, combining organoid technology with gene editing enables the precise correction of genetic disorders in the blood system [33]. Organoids have shown significant potential in the customisation of personalised treatment strategies for solid tumours such as colorectal cancer, liver cancer and gastric cancer [124, 125]. In haematology, bone marrow organoid models have been successfully developed to support the in vitro growth of patient‐derived malignant tumour cells. These models simulate the bone marrow microenvironment, providing an ideal platform for testing and optimising personalised treatment plans [41]. As technology advances, organoid technology is expected to be widely applied in the treatment of haematologic diseases, significantly improving treatment efficacy and safety, particularly in drug screening and personalised immunotherapy [124] (Figure 4).

## 5 Challenges

### 5.1 Technical Limitations

Despite the immense potential of organoid technology in haematologic research, several technical limitations remain. Studies showed that the current organoid technology struggles to fully replicate the complexity and diversity of in vivo tissues, limiting its applications in regenerative medicine and personalised treatment [126]. Additionally, the lack of precise simulation of the microenvironment and matrix during organoid culture poses challenges to organoid growth and functionality. Although organoids partially replicate the structure and function of organs, the absence of a proper microenvironment affects the accuracy of cell interactions and signal transduction [124]. Furthermore, organoids often remain in an embryonic state during long‐term culture, which limits their maturity and applicability in modelling adult diseases and toxicity testing [127]. To overcome the limitations of organoid technology, researchers are continually developing improvement strategies. For instance, engineered scaffold materials can better control the physical and biochemical properties of organoids, enhancing maturation and proper cell signalling [128]. Adjusting culture conditions and introducing specific growth factors also promote further organoid maturation [129]. Additionally, gene editing techniques like CRISPR allow the precise manipulation of gene expression within organoids, enabling them to more accurately reflect patient‐specific differences [130].

### 5.2 Standardisation and Reproducibility Issues

Standardisation and reproducibility issues with organoid technology remain significant challenges in haematologic research. Although organoids can mimic various functions of human tissues, there are significant differences in reproducibility between laboratories. High heterogeneity and the randomness of self‐organising growth led to variability in organoid‐related experimental results, making data standardisation and inter‐laboratory comparisons challenging [131]. Additionally, to enhance the clinical predictive ability of organoid technology, measurable drug response analysis methods that correlate with patient responses need to be developed; however, current standardised models are still insufficient for this purpose [132]. While patient‐derived organoids showed significant potential in predicting personalised treatment responses, their consistency and reliability in standardised testing still require further validation and improvement [133]. However, research indicates that applying engineering techniques, such as microfluidics, can provide greater precision in organoid growth and morphology control, thereby improving their standardisation and reproducibility [129]. With advancements in technology and the unification of guidelines, it is anticipated that issues of standardisation and reproducibility in organoids will be resolved.

## 6 Future Directions

### 6.1 Application of New Technologies

In organoid research, the application of artificial intelligence (AI) and big data technologies is increasingly becoming a key driver of progress in the field. AI and big data can significantly enhance data processing and analysis efficiency in organoid research, thereby accelerating the pace of new discoveries and applications. AI technology can accurately assess organoid growth and functionality through automated image analysis and pattern recognition, significantly reducing human error and workload [124]. Combined with big data analysis, researchers can better understand organoid performance under various experimental conditions, optimise culture conditions and improve reproducibility and standardisation [129]. Additionally, the advantages of big data technology in handling large volumes of experimental data enable cross‐laboratory collaboration and data sharing, thereby advancing the overall progress in organoid research [126]. Excitingly, AI and big data technologies can better simulate and predict organoid responses under different treatment conditions, providing strong support for personalised therapy and new drug development [134]. Building on this, the introduction of bioreactors and semi‐automated cell culture devices has further advanced organoid research. Bioreactors significantly enhance organoid maturation and functionality by precisely controlling culture conditions such as oxygen, nutrients and mechanical stimuli [135]. Additionally, the application of microfluidic technology has made organoid cultures more stable and controllable, particularly excelling in long‐term and large‐scale experiments [136]. Semi‐automated cell culture devices improve experiment reproducibility and standardisation by minimising manual intervention and optimising operational workflows; these systems efficiently handle large sample volumes while enabling real‐time data monitoring and analysis [137, 138]. The integration of these technologies not only boosts the efficiency of organoid research but also opens new opportunities for interdisciplinary collaboration and personalised therapies (Figure 4).

### 6.2 Interdisciplinary Collaboration

The complexity and diversity of organoid technology necessitate interdisciplinary collaboration during research and application to overcome the limitations of single disciplines and drive technological advancement. Recently, the collaborative advancement of fields such as bioengineering, computer science and clinical medicine has significantly improved organoid technology. By integrating bioengineering techniques, it is possible to more precisely control the growth environment and morphology of organoids, enhancing their physiological relevance and application prospects [131]. The integration of genetic editing tools and microfluidic systems in organoid research is also crucial for improving organoid growth and functionality, providing a more stable platform for disease modelling and drug screening [139]. Furthermore, interdisciplinary collaboration can facilitate the translation between clinical and basic research, accelerating the development of innovative treatment solutions [140]. In summary, interdisciplinary collaboration not only drives technological progress but also promotes the standardisation and widespread application of organoid technology through knowledge sharing and cross‐disciplinary communication [126].

## 7 Conclusion

As an innovative biomedical tool, organoid technology is profoundly transforming the landscape of haematologic research. By highly mimicking the 3D microenvironment in vivo, organoid technology provides unprecedentedly precise models for exploring haematopoiesis mechanisms and complex cell interactions. It also demonstrates substantial potential in drug screening, toxicity testing, and disease modelling. Particularly, organoid technology brings a new hope for personalised and precision medicine, enabling more effective research on disease mechanisms and optimisation of treatment strategies.

Despite this, organoid technology still faces challenges such as technical limitations, standardisation and reproducibility on its path to development. However, with the deep integration of AI and big data technologies and the ongoing advancement of interdisciplinary collaboration, these challenges are expected to be effectively addressed.

Looking ahead, organoid technology is set to play an increasingly pivotal role in haematologic research and clinical treatment. Through ongoing refinement and innovation, organoid technology will make even more outstanding contributions to human health.

## Author Contributions

Liangzheng Chang wrote the manuscript. Lu Li was responsible for the diagramming. Liuliu Yang, Hui Cheng and Yuling Han was in charge of literature search and manuscript correction.

## Conflicts of Interest

The authors declare no conflicts of interest.

## Data Availability

Data sharing is not applicable to this article as no new data were created or analyzed in this study.
